# The relationship between shifts in the rhizosphere microbial community and root rot disease in a continuous cropping American ginseng system

**DOI:** 10.3389/fmicb.2023.1097742

**Published:** 2023-02-14

**Authors:** Yan-Meng Bi, Xi-Mei Zhang, Xiao-Lin Jiao, Jun-Fei Li, Na Peng, Gei-Lin Tian, Yi Wang, Wei-Wei Gao

**Affiliations:** ^1^Institute of Medicinal Plant Development, Chinese Academy of Medical Sciences and Peking Union Medical College, Beijing, China; ^2^School of Environmental and Municipal Engineering, Tianjin Chengjian University, Tianjin, China; ^3^School of Biology and Brewing Engineering, Taishan University, Tai'an, Shandong, China; ^4^Biomedicine School, Beijing City University, Beijing, China; ^5^College of Agricultural and Biological Engineering, Heze University, Heze, Shandong, China

**Keywords:** root rot disease, continuous cropping, microbial community, American ginseng, chemical properties

## Abstract

The root rot disease causes a great economic loss, and the disease severity usually increases as ginseng ages. However, it is still unclear whether the disease severity is related to changes in microorganisms during the entire growing stage of American ginseng. The present study examined the microbial community in the rhizosphere and the chemical properties of the soil in 1–4-year-old ginseng plants grown in different seasons at two different sites. Additionally, the study investigated ginseng plants' root rot disease index (DI). The results showed that the DI of ginseng increased 2.2 times in one sampling site and 4.7 times in another during the 4 years. With respect to the microbial community, the bacterial diversity increased with the seasons in the first, third, and fourth years but remained steady in the second year. The seasonal changing of relative abundances of bacteria and fungi showed the same trend in the first, third, and fourth years but not in the second year. Linear models revealed that the relative abundances of *Blastococcus, Symbiobacterium, Goffeauzyma, Entoloma, Staphylotrichum, Gymnomyces, Hirsutella, Penicillium* and *Suillus* spp. were negatively correlated with DI, while the relative abundance of *Pandoraea, Rhizomicrobium, Hebeloma, Elaphomyces, Pseudeurotium, Fusarium, Geomyces, Polyscytalum, Remersonia, Rhizopus, Acremonium, Paraphaeosphaeria, Mortierella, and Metarhizium* spp. were positively correlated with DI (*P* < 0.05). The Mantel test showed that soil chemical properties, including available nitrogen, phosphorus, potassium, calcium, magnesium, organic matter, and pH, were significantly correlated to microbial composition. The contents of available potassium and nitrogen were positively correlated with DI, while pH and organic matter were negatively correlated with DI. In summary, we can deduce that the second year is the key period for the shift of the American ginseng rhizosphere microbial community. Disease aggravation after the third year is related to the deterioration of the rhizosphere microecosystem.

## 1. Introduction

Plant-associated microorganisms play important roles in plant growth, nutrition, and resistance to biotic and abiotic stresses (Vandenkoornhuyse et al., 2015; Hu et al., 2018). Plant rhizospheres provide a rich environment in which diverse microbial communities, including plant-beneficial microbes and pathogenic microbes, coexist (Berendsen et al., 2012; Trivedi et al., 2020; Xia et al., 2021). It is believed that the plant selects its microbial partners through the influence of its rhizodeposits (Sasse et al., 2018). Thus, different plant species support host-specific microbial communities when grown on the same soil (Garbeva et al., 2008; Berg and Smalla, 2009; Xia et al., 2022).

For the past several years, the use of molecular approaches based on high-throughput sequencing has dramatically extended our knowledge of the plant rhizosphere microbial community and revealed the relationship between plant disease and its microbiome. A previous study on the microbiome of *Arabidopsis thaliana* indicated that plants could specifically recruit a group of resistance-inducing and growth-promoting beneficial microbes upon pathogen infection (Berendsen et al., 2018). Shen et al. (2019) demonstrated that biofertilizer application and fumigation could reduce banana Panama disease by establishing a beneficial soil microbiome. Wang et al. (2020) reported that no-tillage and residue management influenced the composition of the soil microbial community and increased the risk of maize root rot. Chen et al. (2020) studied the rhizosphere soil of a 12-year cropping strawberry and found that physicochemical properties, the abundance of key microorganisms, and some phenolic acids accumulated significantly, which might lead to the disease under a continuous cropping system. However, how the specific microbial community of perennial crops forms little by little is still not fully understood. Perennial crops have their particularities, and many perennial plants are valuable economic crops, but perennial plants' diseases usually worsen over years of cultivation (Li et al., 2020; Moore et al., 2022). It is unclear whether the aggravation of plant disease during the development stages was associated with the succession of its rhizosphere microbial community, though. The upshot of this better understanding will substantially impact various research and applications about soil microbial ecology and plant disease. American ginseng (*Panax quinquefolius* L.) is a perennial plant that is well-known globally for its eutherapeutic effect on some diseases (Sen et al., 2012; Singh et al., 2017). In agricultural practice, American ginseng is continuously cultivated for 3 or 4 years before harvest. During the growth of American ginseng, the root rot diseases caused by pathogens reduced the products and quality severely (Yang et al., 2009; Farh et al., 2018), and these diseases became more and more severe over the years of cultivation.

This study aimed to investigate the succession of bacterial and fungal communities and their relationships with root rot disease in American ginseng over years of cultivation. In addition, chemical properties were detected in a correspondence soil sample to analyze their correlation with microbial communities and disease. The results obtained from this study will be valuable for gaining insight into the impact of different cropping systems on soil micro-ecology, which can aid in the cultivation of perennial crops and enhance sustainable development in the medicinal industry.

## 2. Materials and methods

### 2.1. Sample processing

The sample sites were in Wendeng Dist., the Wehai City of Shandong Province, one of China's main American ginseng-producing regions. In the first year of the study, 7.5 metric tons of organic fertilizer (with an organic matter content >80%) were applied per hectare. In the following years, increasing amounts of compound fertilizer were applied per hectare: 150–200 kg in the second year, 300–400 kg in the third year, and 450–600 kg in the fourth year. The compound fertilizer used had a nitrogen, phosphorus, and potassium content >16%. This region has a northern temperate marine monsoon climate and receives an annual precipitation of ~762 mm. The plow layer in the plantation consists of gray-brown soil. In 2017, it was decided to directly sow American ginseng for 1 to 4 years in adjacent charmilles of Xishuipo Village, Dashuipo Town (122°14′07″E, 37°10′48″N, marked as Site I). For each ginseng age, four sampling points (replicates) of about 10 m^2^ were chosen and marked for subsequent sampling.

During the spring (late May), summer (late July), and autumn (late September), at each sampling location, half-row ginsengs (6–10 individuals) were collected, and the rhizosphere soil of these ginsengs was thoroughly mixed. Some amounts of soil was stored at −80°C for DNA extraction, while the rest was air-dried for chemical analysis. The ginseng from each site collected in the summer and autumn was cleaned to calculate the disease index (DI). In the summer (July 20th) and autumn (September 20th) of 2018, the experiment was repeated in Liujiatuan Village of Zetou Town (121°51′44″E, 37°03′14″N, referred to as Site II), another town of Wendeng District. In total, 80 = 4 replications × 4 ages × [3 seasons (of 2017, Site I) + 2 seasons (of 2018, Site II)] soil samples were obtained (Supplementary Table S1).

### 2.2. Disease index calculations

The root rot disease index (DI) was estimated by dividing the number of diseased ginseng roots by the total number of plants investigated. The severity of root disease observed in the pot experiment was determined by the presence of surface lesions, which were quantified on a scale from 0 to 4, with 0 representing no lesions and 1, 2, 3, and 4 standing for lesions that are <10%, 10%−33%, 33%−67%, and larger than 67% of the total area of the root. The severity of the disease at one sampling site was recorded as the DI, which was calculated as follows:


$$\begin{array}{l} \text{DI}=\frac{\sum \left(Si\times Xi\right)}{4\times N}\times 100\; \end{array}$$

Si is the severity rating, Xi is the number of roots with the corresponding severity rating, and N is the total number of roots in one sampling site (Jiao et al., 2019).

### 2.3. Soil chemical properties

The soil chemical properties, including available nitrogen (AN), available phosphorus (AP), available potassium (AK), organic matter (OM), pH, exchangeable calcium, and magnesium (E-Ca and E-Mg), were measured with the alkali hydrolysis diffusion method, the NaHCO_3_ extraction method, the NH_4_OA_C_ extraction method, the potassium dichromate volumetric method, the pH meter, and the atomic absorption spectrophotometry, respectively, as described by Bao (2000).

### 2.4. DNA extraction, PCR, and high-throughput sequencing

Soil microbial DNA was extracted from 0.4 g soil with the PowerSoil DNA Isolation Kit (Mobio Laboratories Inc., Carlsbad, CA, USA) according to the manufacturer's instructions. The bacterial universal V3-V4 region of the 16S rRNA gene was amplified with amplicon PCR forward primer 338F (5′-ACTCCTACGGGAGGCAGCAG-3′) and reverse primer 806R (5′-GGACTACCAGGGTATCTAAT-3′) (Zheng et al., 2017). The fungal universal ITS1 region was amplified with the amplicon PCR forward primer (5′-CTTGGTCATTTAGAGGAAGTAA-3′) and reverse primer (5′-TGCGTTCTTCATCGATGC-3′) (Mukherjee et al., 2014). Three PCR products per sample were pooled, purified, and quantified by real-time PCR. Parallel-tagged sequencing was performed on an Illumina MiSeq platform (Allwegene, Beijing, China) according to standard protocols (Edgar, 2013; Zhang et al., 2018). Specifically, split reads were merged using FLASH (Magoc and Salzberg, 2011), where forward and reverse reads had overlapping base lengths ≥10 bp and were sorted into each sample by the unique barcodes with QIIME (Caporaso et al., 2010). The sequences with a quality score below 20 contained ambiguous bases or did not exactly match the primer sequences, and barcode tags were removed to become raw data (Wang et al., 2019). Chimeras were removed with USEARCH against the Gold and UNITE reference databases, and sequences shorter than 200 bp were removed to become clean data. The high-quality sequences were clustered into operational taxonomic units (OTUs) at a threshold of 97% similarity using the UPARSE pipeline (Edgar, 2013). Singletons that occurred only one time in the entire data set were removed from subsequent analyses to reduce the overprediction of rare OTUs (Jiao et al., 2019). The representative OTU sequences were aligned and annotated using the Ribosomal Database Project (RDP) for 16S and Unite for ITS (Shen et al., 2019). The datasets generated for this study can be found in NCBI, and the BioProject accession numbers are PRJNA890432 for bacteria and PRJNA890915 for fungi.

### 2.5. Statistical analyses

DI was expressed as means and standard deviation of four replicates. ANOVA was performed with SPSS 19.0 (SPSS Inc., Chicago, IL, USA), and significant differences among groups were determined at the *P* < 0.05 level according to the Duncan multiple range test. Other statistical analyses were based on R programs (v3.2.2; http://www-r-project.org/). The alpha (α) diversity indexes Chao1 and Shannon were calculated to assess the microbial abundance and diversity, and the differences among different groups were tested by Duncan's multiple range test. Microbial beta-diversity was quantified with two axes of a non-metric multidimensional scaling (NMDS) analysis of Bray–Curtis dissimilarities in the OTUs community matrix using the “vegan” package in R. The alluvial figures over time for bacterial phylum and fungal class were based on the “ggalluvial“ package in R, and Proteobacteria was divided into Alpha-, Beta-, Delta-, and Gamma-Proteobacteria classes due to the high relative abundance of Protecobacteria. To obtain the biomarkers of microbial taxa across American ginseng residence time in the field, we used the Random Forests approach provided by Zhang J. et al. (2018) to regress the relative abundances of bacterial and fungal taxa at the genus level against American ginseng residence time in the field. We showed the obtained biomarkers with the “phetamap” package in R. Linear models for the relationships of microbial indicators with DI and the relative importance of each of the predictors in this model was also tested after stepwise model selection using stepAIC in R to select the best explanatory power. The Mantel test was used to identify correlations between microbial composition and soil chemical properties, and perMANOVA was used to test the effects of American ginseng over years of cultivation and seasons on soil chemical properties (Shen et al., 2019). Bray–Curtis distance matrices and Euclid distance matrices were used for microbial composition and soil environmental factors, respectively (Jiao et al., 2016). The ordination diagram was done based on original data of soil chemical properties, DIs of American ginseng, and relative abundances of disease-related microorganisms, using Canoco 4.5 software (Microcomputer Power, Ithaca, NY, USA) with the method described by Bi et al. (2018).

## 3. Results

### 3.1. Root disease indices of American ginseng

**Figure 1:**
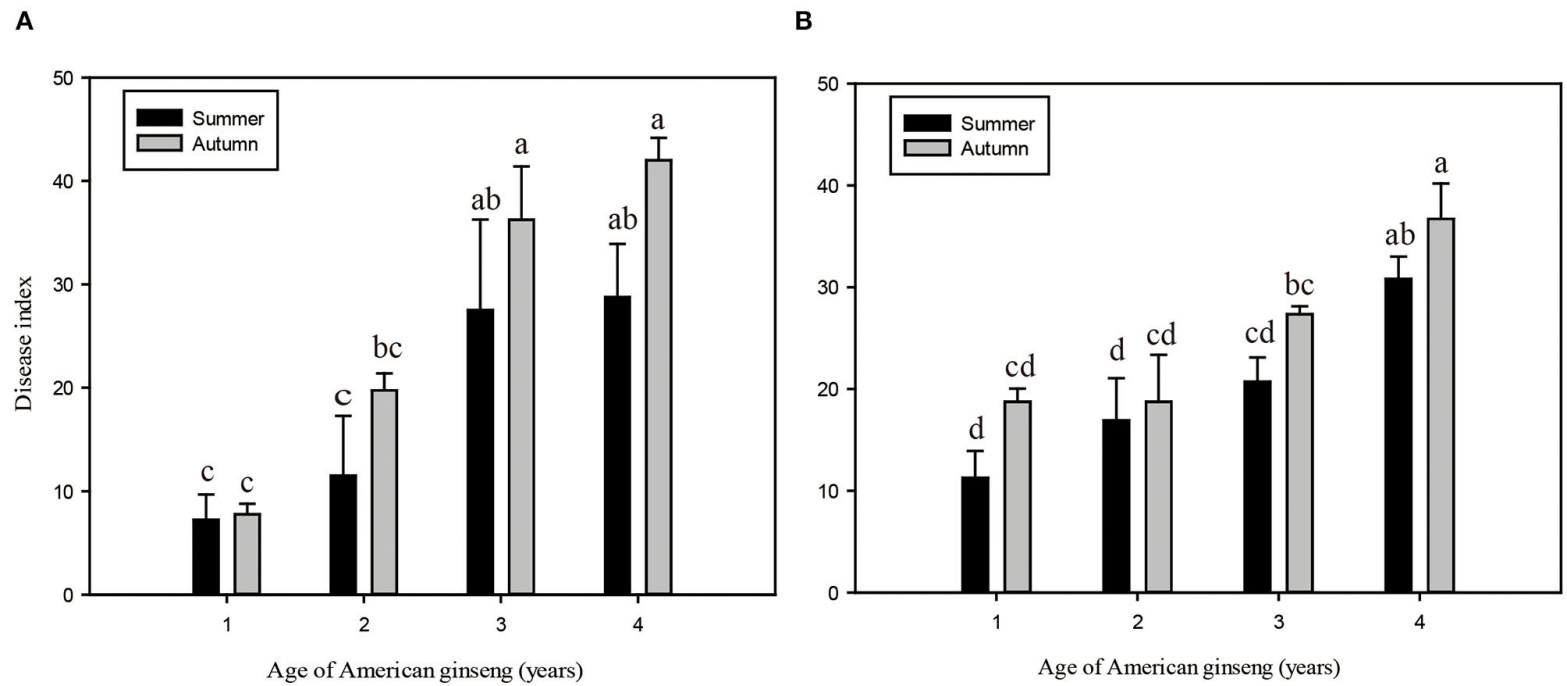
Root disease index of American ginseng of different ages in summer and autumn of Dashuipo in 2017 **(A)** and Zetou in 2018 **(B)**. Error bars represent standard errors of four replicates, and different letters indicate a significant difference among different ages of ginseng (*P* < 0.05, according to Duncan's multiple range test).

During the four years of the growing phase of American ginseng, the roots gradually became larger (Supplementary Figure S1A), and the root weight of ginseng increased from 0.36 ± 0.14 g to 17.81 ± 7.46 g (Supplementary Figure S1B). Meanwhile, the root rot disease of American ginseng exhibited an aggravating trend with ginseng ages in both sites (Figures 1A, B). The disease indices of ginseng were higher in autumn than in summer for the corresponding ages, though no significant difference was found. In Site I, the disease indices of 3- and 4-year ginsengs were significantly higher (*P* < 0.05) than those of 1- and 2-year ginsengs in the corresponding seasons, while in Site II, 4-year ginsengs exhibited a significantly higher DI than 1-, 2-, and 3-year ginsengs. Specifically, the disease indices ranged between 7.3 and 42.0 in Site I and between 11.3 and 36.7 in Site II.

### 3.2. Dynamic of rhizosphere microbiota overtime during the 1-year to 4-year growth of American ginseng

Across all the samples, we obtained a total of 3,817,186 and 4,471,469 high-quality 16S and ITS sequences, which were respectively grouped into 9,704 and 4,415 OTUs when using the 97% sequence similarity cutoff. The most abundant bacterial phylum were Proteobacteria (50.8%), Acidobacteria (15.5%), Actinobacteria (11.4%), and Chloroflexi (6.3%), while fungal sequences were primarily composed of the phylum Ascomycota (62.1%), Mortierellomycota (16.7%), and Basidiomycota (11.3%). According to the rarefaction and species accumulation curves, it can be inferred that the sequencing depth and sample amount are enough for subsequent analysis (Supplementary Figure S2).

**Figure 2:**
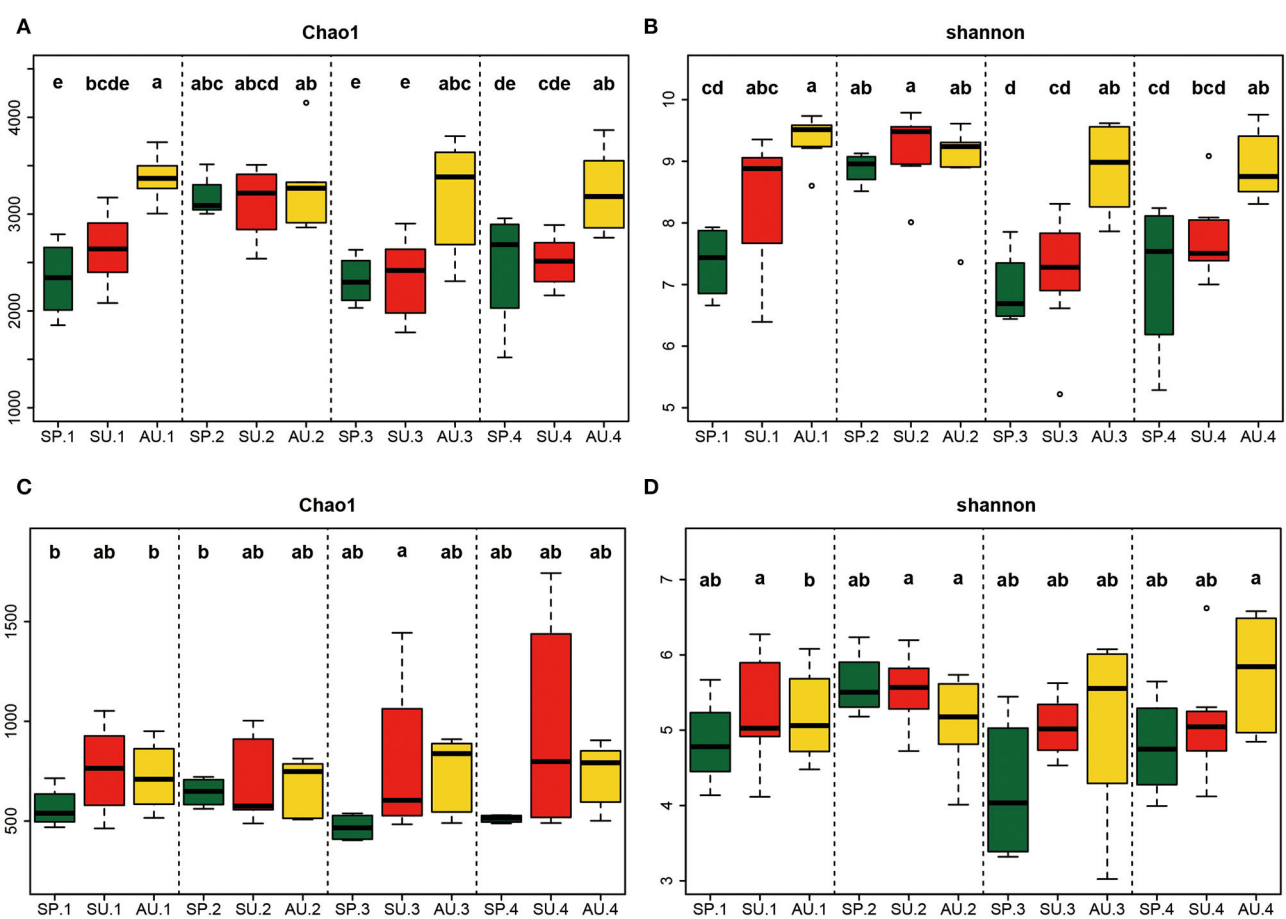
Alpha-diversity of microorganisms during the growing stages of American ginseng. Chao1 of bacteria **(A)**, Shannon of bacteria **(B)**, Chao1 of fungi **(C)**, and Shannon of fungi **(D)**. Thick horizontal bars show the median. The upper and lower “hinges” correspond to the 25th and 75th percentiles, and whiskers extend from the hinge to the highest (or lowest) value that is within 1.5 × interquartile range (IQR) of the hinge. Boxes that do not share the same letter indicate a significant difference (*P* < 0.05, according to Duncan's multiple range test).

For bacteria at the two sites, the Shannon indices increased with season changes in the first year, peaked in the autumn, and then maintained a high level in the second year. In the third and fourth years, the indices exhibited a similar trend to the first year (Figures 2A, B). Besides, in spring and summer, the Shannon indices for 2-year American ginseng cultivated soil were compared to the other 3 years. However, the indices for autumn soil over the 4 years were similar. The fungi Shannon indices did not change significantly between years, and the trend was similar with bacteria (Figures 2C, D).

**Figure 3:**
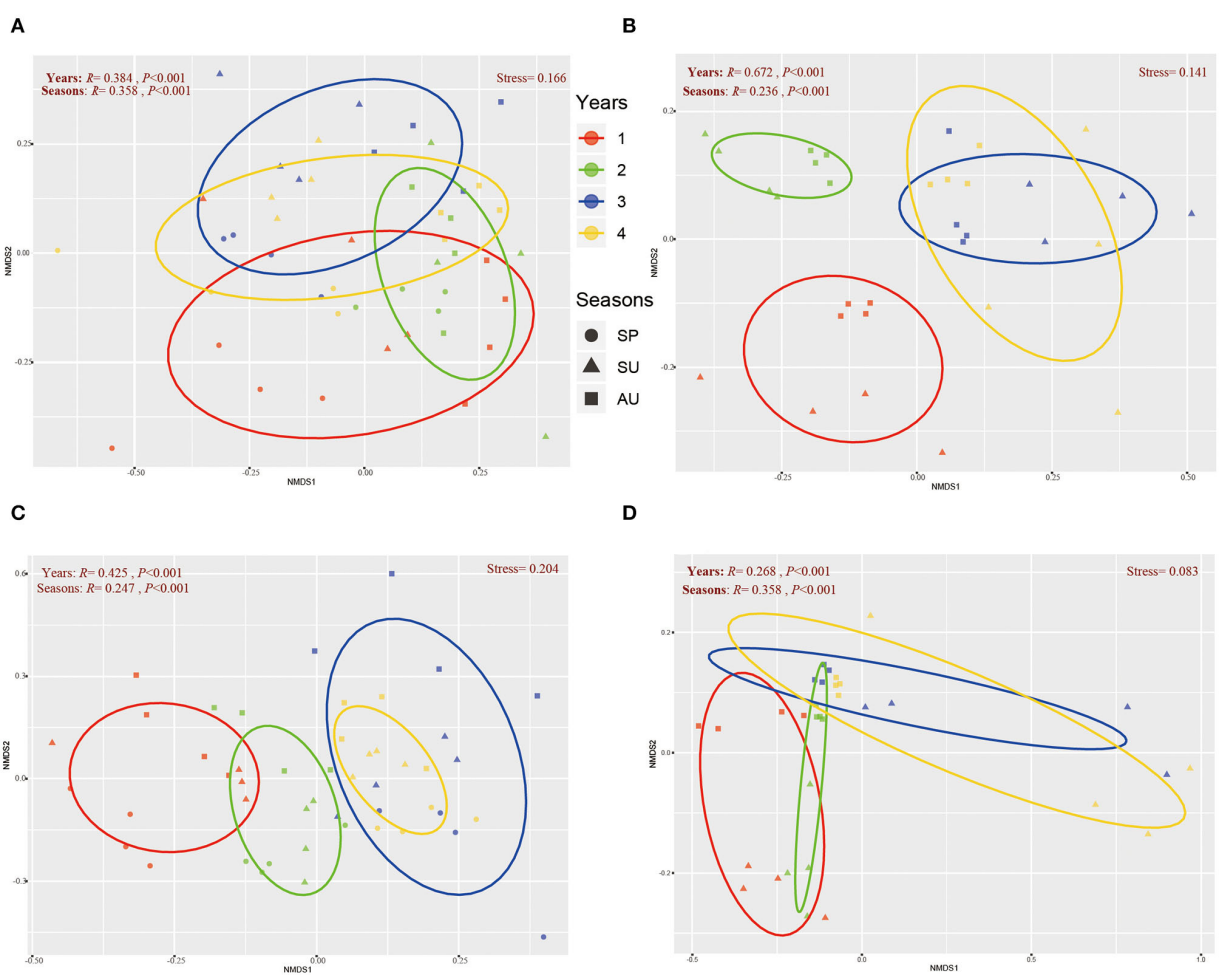
The general pattern of microbial beta-diversity in the soil of three seasons during four years. NMDS showed the structure of the bacterial community of Site I **(A)** and Site II **(B)**, and the fungal community of Site I **(C)** and Site II **(D)**. Similarity values among the samples of different seasons (“Seasons”) during 1- to 4-year (“Years”) were examined *via* the ANOSIM test, which are shown in each plot. 60% confidence ellipses were shown around the samples grouped based on different ages of American ginseng (years).

The NMDS revealed that the soil microbial community of American ginseng rhizosphere soil exhibited a gradient change among seasons during 4 years of growth (Figure 3), with significant differences being found at taxonomic levels (ANOSIM test). The differences in bacterial communities among years were larger than those between seasons at both sites (Figures 3A, B), indicating that soil bacterial communities were more influenced by years of cultivation than seasons. With respect to the fungal community, the differences among years were larger than seasons at Site I (Figure 3B) but were smaller than seasons at Site II (Figure 3D). Moreover, we found that the microbial communities of 3- and 4-year American ginseng rhizosphere soil at both sites were so similar that they could not be separated in the NMDS plots. However, the microbial communities of 1-year ginseng rhizosphere soil were far from those of 3- and 4-year ginseng, while the microbial communities of 2-year ginseng rhizosphere soil sat between those of 1-, 3-, and 4-year ginseng rhizosphere soil.

**Figure 4:**
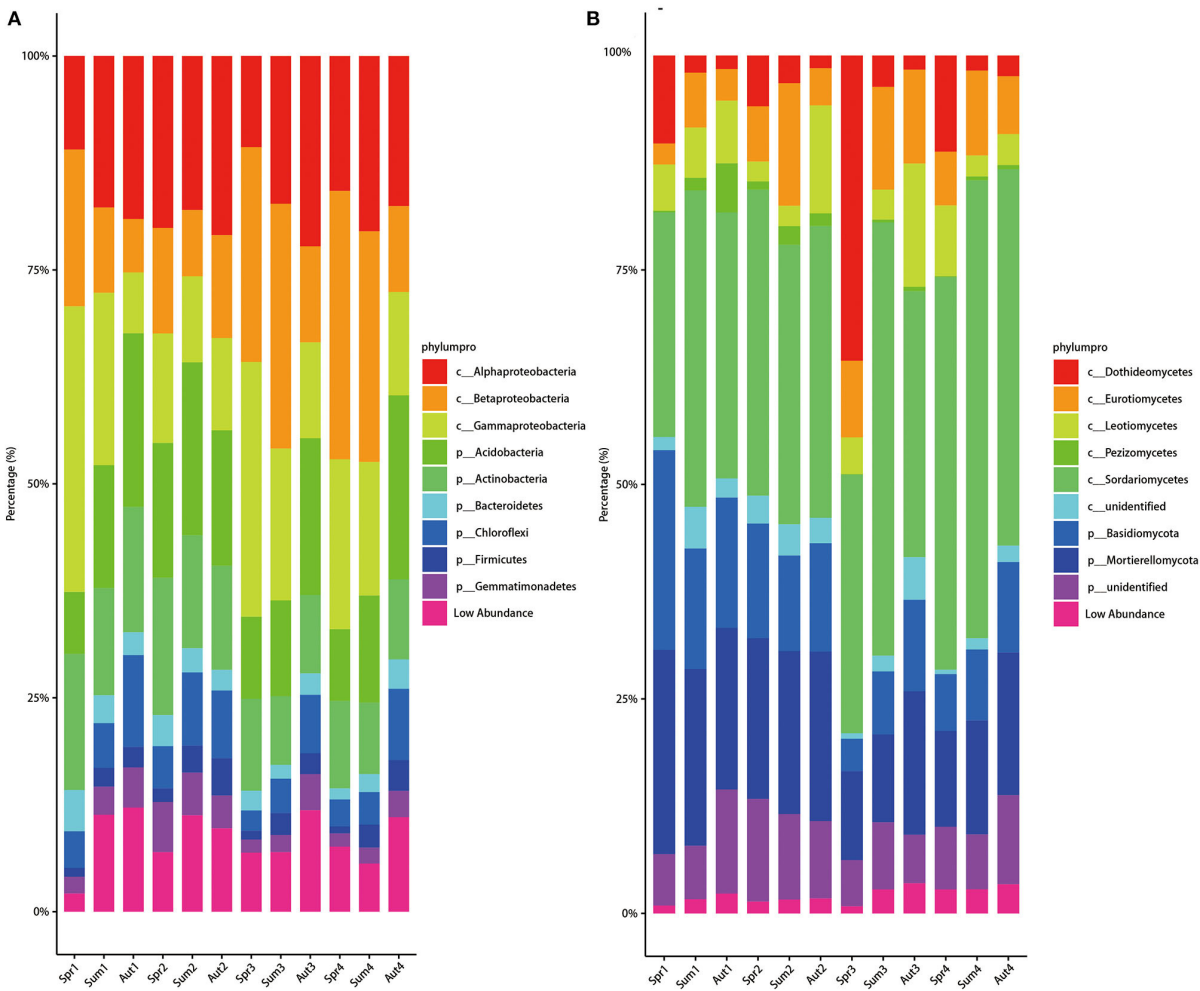
Average relative abundances change over time of bacterial phylum **(A)** and fungal class **(B)**. Alluvial figures were plotted based on 80 samples of the two sites.

The relative abundances of bacterial phyla exhibited a distinct cyclic variation with seasons during the 4 years except for the second year (Figure 4A). From spring to autumn, the relative abundance of Acidobacteria increased while that of Gammaproteobacteria decreased dramatically in 1-, 3-, and 4-year soils. For 2-year-old soil, among different seasons, the bacterial composition kept steady. Compared with the bacterial phylum, the fungal composition fluctuated slightly in class level among different seasons and years, except for the samples of spring in the 3-year soil (Figure 4B). In spring in the 3-year soil, the relative abundance of Dothideomicetes was higher, while the relative abundance of Mortierellomycetes was lower than the other groups. Furthermore, we discovered that Dothideomicetes was most abundant in the spring of all 4 years of soil. Besides, the relative abundance of Tremellomycetes in the three seasons' soil during 1 and 2 years the soil was higher than that in 3 and 4 years.

### 3.3. Specific taxa of the root microbiota are associated with residence time

**Figure 5:**
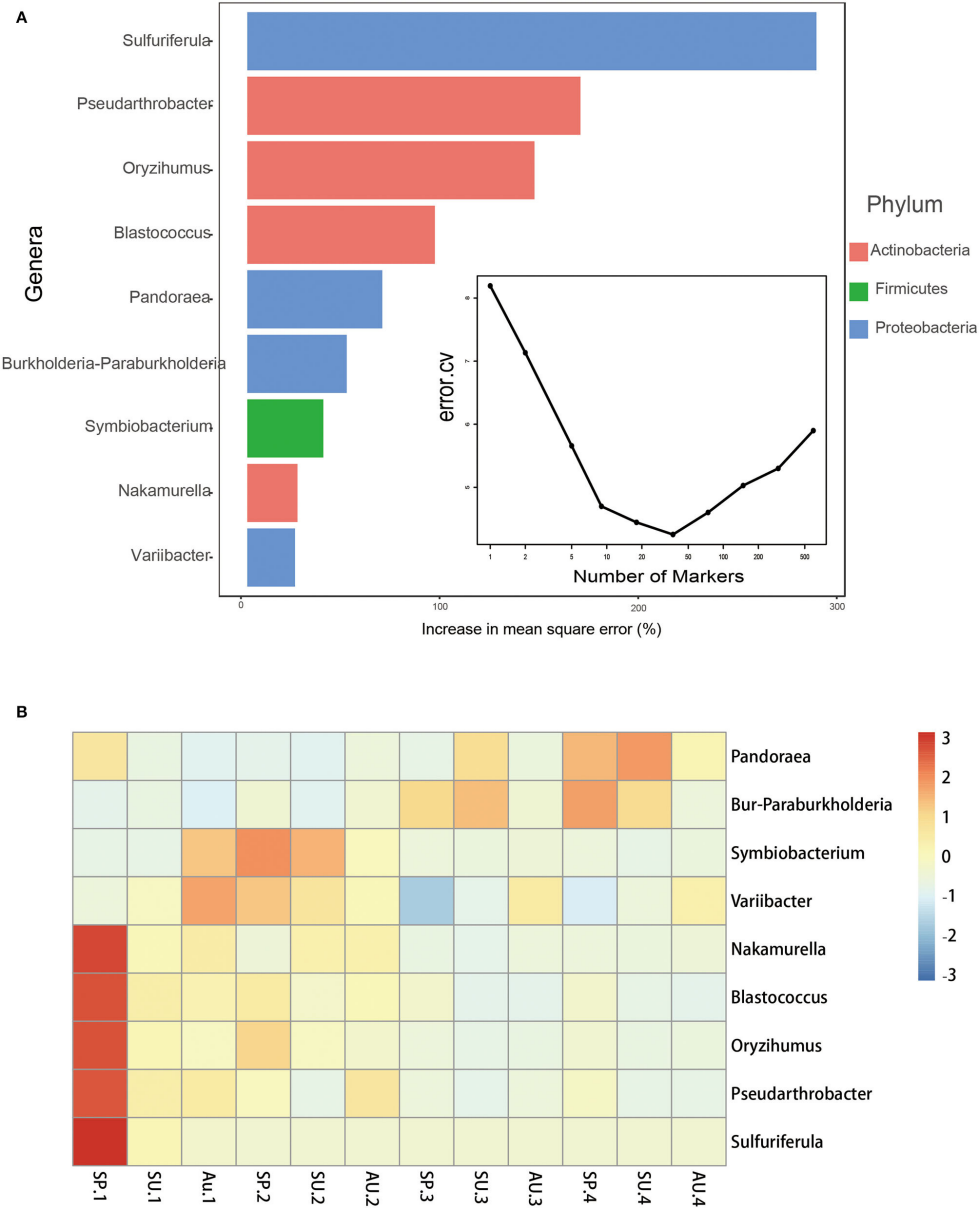
Bacterial taxonomic biomarkers of American ginseng cultivated time in fields. **(A)** The top nine biomarker bacterial genera were identified by applying Random Forests regression of their relative abundances in soil against American ginseng years of cultivation and seasons in the field. Biomarker taxa are ranked in descending order of importance to the accuracy of the model. **(B)** A heat map showing the relative abundances of the top nine predictive biomarker bacterial genera.

Based on the cross-validation result of the rfcv() function in the R package “randomForest,” nine bacterial genera were screened as biomarkers (Figure 5A). The heatmap showed that the relative abundance of *Sulfuriferula, Pseudarthrobacter, Oryzihumus, Blastococcus, Nakamurella, Variibacter*, and *Symbiobacterium* decreased over residence time, while that of *Pandoraea* and *Burkholderia-Paraburkholderia* increased. Based on how these bacteria are classified at the phylum level, the number of biomarkers that belong to Actinobacteria and Firmicutes has decreased over time, while the number of biomarkers that belong to Proteobacteria has both increased and decreased (Figure 5B).

With the same method, 22 fungal genera were screened as biomarkers (Supplementary Figure S3A). The relative abundances of *Staphylotrichum, Gymnomyces, Solicoccozyma, Cystodendron, Dioszegia, Lipomyces, Byssocorticium, Stilbella, Pseudaleuria*, and *Trechispora* of the first year were higher than those of the last 3 years and exhibited a decreasing trend over the years; those of *Hebeloma, Remersonia, Chaetomidium, Chaetomium, Polyscytalum*, and *Suillus* increased and reached a peak in the second year and then decreased; while those of *Fusarium, Pseudeurotium, Geomyces, Elaphomyces, Mortierella*, and *Sordaria* were higher in the last 2 years (Supplementary Figure S3B) when the root rot was severe, their abundances decreased. *Mortierella* belongs to Mortierellomycota, while the rest of the biomarkers belong to Ascomycota and Basidiomycota.

In total, 14 fungi genera were isolated and identified in the diseased root, with Fusarium accounting for the highest proportion (63.9%) and *Trichoderma* accounting for the lowest (12.0%), and the proportion of *Fusarium* increased while that of *Trichoderma* decreased with ginseng age. In addition, *Rhizopus, Plectosphaerella, Mortierella, Alternaria, Zalerion, Rosellinia, Rhizoctonia, Pythium, Penicillium, Paraphaeosphaeria, Mucor*, and *Chaetomium* were also identified, and a few isolated fungi were not identified (Supplementary Figure S4).

### 3.4. Relationship between microbial indicators and DI

To study the relationship between DI and microbial indicators, two linear models, bacterial indicators and fungal indicators, were constructed, respectively. Bacterial indicators included Chao1, Shannon, NMDS1, and NMDS2, the screened biomarkers of time (Figure 5). The retained indicators after stepwise selection are shown in Table 1, and the proportion of variance explained by the model was 46.2%. Of the retained indicators, the relative abundances of *Pandoraea, Rhizomicrobium*, and bacterial Chao1 were positively correlated with DI, while those of *Blastococcus* and *Symbiobacterium* were negatively correlated with DI (*P* < 0.05, Table 1). Fungal indicators included Chao1, Shannon, NMDS1, and NMDS2, the screened biomarkers of time (Supplementary Figure S4), and the fungi isolated from the rotted root of American ginseng (Supplementary Figure S4) were initially selected. The retained indicators after stepwise selection are shown in Table 2, and the proportion of variance explained by the model was 87.5%, which was higher than the model constructed with bacterial indicators and DI, indicating that fungi may play a more important role in the occurrence of root rot disease in American ginseng.

**Table 1:** Linear models (LM) for the relationship of bacterial indicators with American ginseng root rot disease and the relative importance of each of the predictors in the model.

**ID**	**Standard error**	***t***	***P*** **(>|t|)**	**Relative importance**
*Pandoraea*	0.108	3.46	**0.001**	0.374
*Rhizomicrobium*	0.138	2.557	**0.013**	0.354
*Blastococcus*	0.129	−2.583	**0.012**	0.334
*Symbiobacterium*	0.104	−2.98	**0.004**	0.311
Chao1	0.115	2.273	**0.027**	0.261
*Sulfuriferula*	0.121	−1.864	0.068	0.226
NMDS2	0.144	1.42	0.161	0.204
Model summary: R^2^ = 0.462, AIC = −24.64, *P* < 0.001				
Proportion of variance explained by model: 46.2%				

The bold value represents the P-value is lower than 0.05 level from the ANOVA result.

**Table 2:** Linear models (LM) for the relationship of fungal indicators with American ginseng root rot disease and the relative importance of each of the predictors in the model.

**ID**	**Standard error**	***t***	***P*** **(>|*t*|)**	**Relative importance**
*Hebeloma*	1.048	3.444	**0.002**	3.61
*Elaphomyces*	0.537	3.724	**0.001**	1.999
*Goffeauzyma*	0.485	−3.224	**0.003**	1.564
*Entoloma*	0.297	−3.855	**0.001**	1.145
*Pseudeurotium*	0.371	2.513	**0.017**	0.933
NMDS1	0.249	3.115	**0.004**	0.775
NMDS2	0.126	6.071	**<0.001**	0.763
Chao1	0.23	2.861	**0.007**	0.658
*Fusarium*	0.129	4.289	**<0.001**	0.553
*Geomyces*	0.197	2.763	**0.009**	0.545
*Polyscytalum*	0.219	2.104	**0.043**	0.462
*Staphylotrichum*	0.101	−4.528	**<0.001**	0.457
*Remersonia*	0.091	4.885	**<0.001**	0.446
*Gymnomyces*	0.141	−3.075	**0.004**	0.432
*Rhizopus*	0.172	2.439	**0.020**	0.419
*Hirsutella*	0.159	−2.495	**0.018**	0.396
*Acremonium*	0.073	4.525	**<0.001**	0.331
*Penicillium*	0.104	−3.165	**0.003**	0.33
*Tomentella*	0.17	−1.919	0.064	0.327
*Paraphaeosphaeria*	0.116	2.441	**0.020**	0.283
*Mortierella*	0.091	2.681	**0.011**	0.245
*Metarhizium*	0.116	2.073	**0.046**	0.24
*Trichophyton*	0.178	1.281	0.209	0.228
*Talaromyces*	0.169	−1.272	0.212	0.215
*Trichoderma*	0.144	1.418	0.166	0.204
*Suillus*	0.068	−2.745	**0.010**	0.186
*Trechispora*	0.097	−1.484	0.147	0.144
*Plectosphaerella*	0.074	1.316	0.197	0.097
Model summary: *R*^2^ = 0.875, AIC = −73.41*, P* < 0.001				
Proportion of variance explained by model: 87.5%				

The bold value represents the P-value is lower than 0.05 level from the ANOVA result.

### 3.5. Relationship between soil chemical properties and community composition

Soil chemical properties differed significantly among different samples. According to the multi-factor analysis of the general linear model, years of cultivation influenced pH, OM, AP, AK, and E-Mg, and sampling seasons influenced AN, AK, E-Ca, and E-mg. Sampling sites influenced pH, OM, AN, AP, AK, and E-Ca (Table 3). In both two sites, the soil is slightly acidic, with the pH at a range of 4.5–5.4. The contents of AN, AK, E-Ca, and E-Mg were higher, while AP was lower in Site I than in Site II. Regarding the over 2 years of cultivation, the pH and AP of the 2-year soil were the highest of the 4 years' soil. No clear trend was found, considering the changes in chemical properties among seasons.

**Table 3:** Soil chemical properties at two sampling sites of different ginseng ages.

**Sample**	**pH**	**OM (%)**	**AN (mg kg^−1^)**	**AP (mg kg^−1^)**	**AK (mg kg^−1^)**	**E-Ca (g kg^−1^)**	**E-Mg (mg kg^−1^)**
D-Spr1	5.04 ± 0.25ab	1.18 ± 0.16bcd	107.91 ± 16.03abc	19.89 ± 12.79b	164.97 ± 93.92c	2.20 ± 0.54ab	198.63 ± 63.61ab
D-Sum1	5.02 ± 0.15ab	1.22 ± 0.28bcd	96.88 ± 3.94bc	21.95 ± 9.04b	180.64 ± 3.78c	1.66 ± 0.50abc	168.38 ± 48.18abc
D-Aut1	5.21 ± 0.14ab	1.33 ± 0.16bcd	86.89 ± 3.42c	25.61 ± 10.49b	157.15 ± 30.85c	0.96 ± 0.42cd	151.27 ± 80.04abc
D-Spr2	5.13 ± 0.21ab	1.84 ± 0.52a	115.40 ± 4.63ab	62.43 ± 19.46a	223.09 ± 34.06bc	2.36 ± 0.08a	190.21 ± 19.50ab
D-Sum2	5.42 ± 0.40a	1.70 ± 0.31ab	93.7 ± 9.29bc	66.32 ± 16.81a	212.68 ± 40.75bc	1.73 ± 0.68abc	155.49 ± 41.62abc
D-Aut2	5.19 ± 0.01ab	1.63 ± 0.19abc	108.80 ± 5.32abc	61.80 ± 7.93a	250.53 ± 1.61bc	0.97 ± 0.12cd	85.01 ± 3.07bc
D-Spr3	4.84 ± 0.24b	0.92 ± 0.15d	89.23 ± 7.24c	32.87 ± 9.60b	211.24 ± 24.37bc	1.92 ± 0.05abc	193.36 ± 13.70ab
D-Sum3	5.01 ± 0.15ab	1.12 ± 0.23bcd	93.84 ± 13.17bc	33.49 ± 10.9b	283.84 ± 45.07b	1.32 ± 0.50bcd	140.12 ± 47.54abc
D-Aut3	4.99 ± 0.13ab	1.13 ± 0.28bcd	126.16 ± 6.87a	31.80 ± 9.48b	374.34 ± 64.95a	0.67 ± 0.22d	106.09 ± 19.65bc
D-Spr4	5.07 ± 0.13ab	1.14 ± 0.22bcd	102.39 ± 11.49bc	48.07 ± 10.62ab	190.02 ± 19.04bc	2.31 ± 0.27a	222.29 ± 40.80a
D-Sum4	5.20 ± 0.27ab	1.08 ± 0.15cd	98.21 ± 12.33bc	49.09 ± 12.9ab	246.60 ± 44.08bc	2.12 ± 0.83ab	203.26 ± 28.62ab
D-Aut4	5.19 ± 0.19ab	1.01 ± 0.18d	114.01 ± 14.22ab	39.82 ± 18.20ab	282.08 ± 41.74b	1.05 ± 0.29cd	132.44 ± 22.59abc
Z-Sum1	4.94 ± 0.19cd	1.17 ± 0.16a	72.92 ± 10.29ab	82.27 ± 36.17a	211.61 ± 53.11ab	0.85 ± 0.07abc	206.90 ± 38.34a
Z-Aut1	5.16 ± 0.06bcd	1.29 ± 0.12a	102.13 ± 17.85a	58.24 ± 7.58ab	137.66 ± 12.93b	1.05 ± 0.18ab	239.24 ± 20.84a
Z-Sum2	5.46 ± 0.12a	0.99 ± 0.15a	78.20 ± 18.37ab	86.90 ± 9.70a	155.15 ± 14.79b	0.78 ± 0.05abc	113.28 ± 8.26b
Z-Aut2	5.19 ± 0.11bcd	1.12 ± 0.07a	81.72 ± 17.39ab	79.89 ± 10.34a	150.71 ± 4.04b	0.62 ± 0.04bc	111.31 ± 6.20b
Z-Sum3	4.81 ± 0.03d	1.13 ± 0.06a	93.96 ± 20.59ab	52.56 ± 6.42ab	253.74 ± 42.12a	0.54 ± 0.19c	122.20 ± 22.66b
Z-Aut3	5.02 ± 0.02bcd	1.00 ± 0.11a	78.22 ± 11.29ab	46.97 ± 5.21b	161.36 ± 17.09b	1.17 ± 0.25a	217.71 ± 36.20a
Z-Sum4	4.97 ± 0.21bcd	1.00 ± 0.22a	65.48 ± 9.42b	44.90 ± 13.12b	176.88 ± 64.03b	0.89 ± 0.42abc	166.61 ± 93.11ab
Z-Aut4	4.86 ± 0.03d	1.01 ± 0.11a	72.71 ± 4.02ab	54.29 ± 13.51ab	179.70 ± 35.46b	0.63 ± 0.20bc	115.35 ± 15.05b
Significant due to							
Years	*	*	NS	*	*	NS	*
Seasons	NS	NS	*	NS	*	*	*
Sites	*	*	*	*	*	*	NS

OM, organic matter; AN, available nitrogen; AP, available phosphorus; AK, available potassium; E-Ca, exchangeable calcium; E-Mg, exchangeable magnesium; NS, not significant different.

^*^Stands for significant different (P < 0.05). Columns that do not share the same letter indicate a significant difference (P < 0.05) according to Duncan's multiple range test.

**Figure 6:**
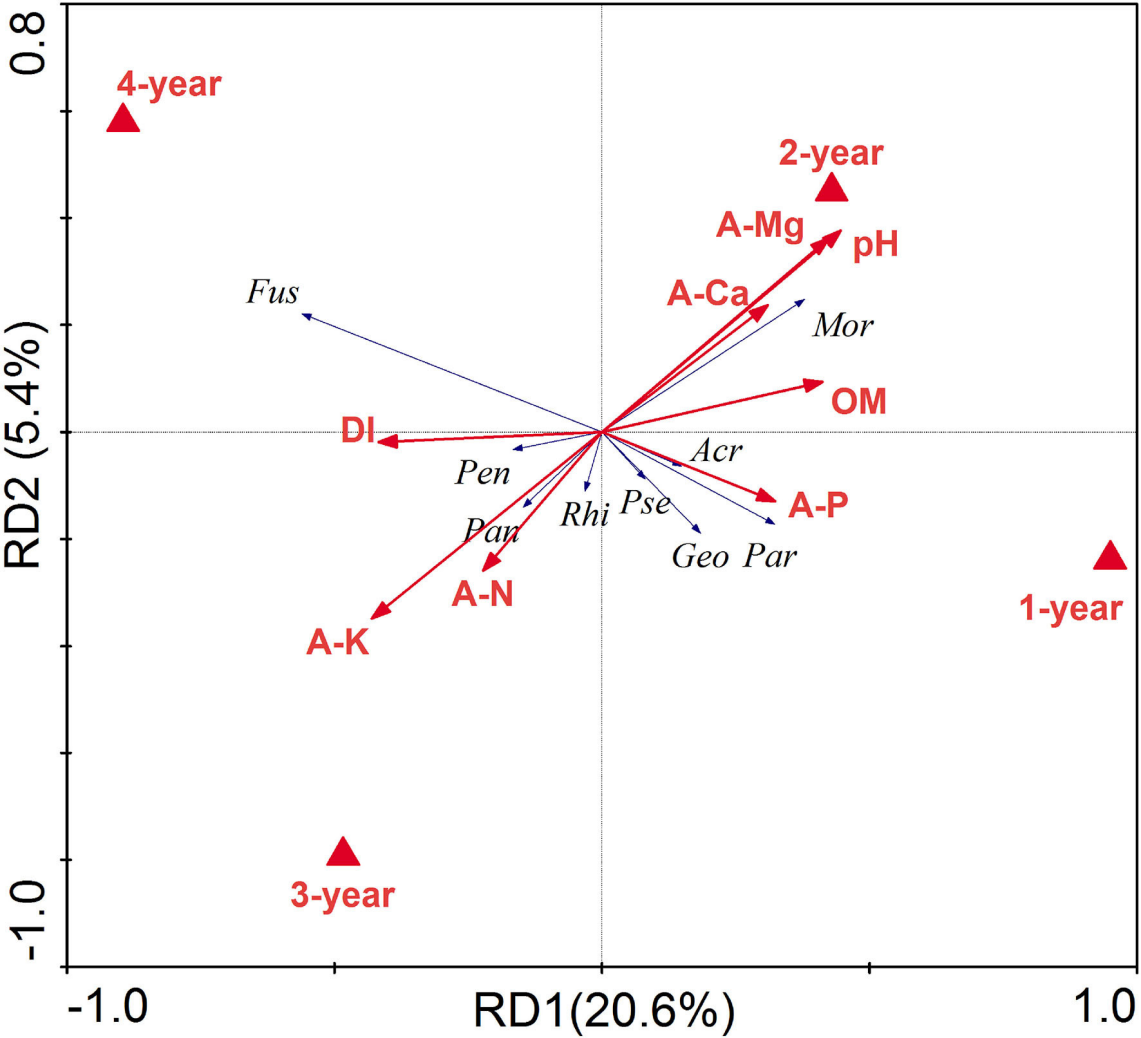
Redundancy analysis (RDA) between soil chemical properties, Disease index (DI), and the relative abundance higher than 1% of microbial genera related with DI. The environmental parameters are represented by red lines, different treatments are solid triangles, and microbial genera are blue lines. *Fus, Fusarium*; *Rhi, Rhizomicrobium*; *Pan, Pandoraea*; *Mor, Mortierella; Pen, Penicillium*; *Geo, Geomyces*; *Acr, Acremonium*; *Pse, Pseudeurotium*; *Rhi, Rhizopus*; *Par*: *Paraphaeosphaeria*.

Soil chemical properties were significantly correlated to both the bacterial (Mantel: *R* = 0.2516, *P* < 0.001) and fungal (Mantel: *R* = 0.3030, *P* < 0.001) (Supplementary Table S2). To disentangle the relationship between soil chemical properties, DI, and bacterial and fungal genera related to DI screened by linear models, RDA was done based on the above indicators (Figure 6). The ordination diagram showed that AK and AN were positively correlated with *Pandoraea* and *Rhizomicrobium*, pH, E-Ca, and E-Mg were positively correlated with *Suillus, Blastococcus, Mortierella*, and *Symbiobacterium*, organic matter was positively correlated with *Symbiobacterium* and *Elaphomyces*, and AP was positively correlated with *Laccaria, Hebeloma, Geomyces, Gymnomyces, Hirsutella, Polyscytalum, Remersonia, Acremonium, Goffeauzyma, Pseudeurotium, Entoloma, Inocybe, Rhizopus* and *Paraphaeosphaeria*. Moreover, the concentrations of AK and AN were positively correlated with DI, while those of AP, E-Mg, E-Ca, organic matter, and pH were negatively correlated with DI. In addition, AK and AN were higher in 3- and 4-year ginseng residence soil, while pH, AP, E-Mg, E-Ca, and organic matter were relatively high in 1- and 2-year ginseng residence soil, indicating that the soil chemical properties affected the occurrence of root rot disease in ginseng.

## 4. Discussion

In our study, the DI became larger with the American ginseng age, which is similar to the study by Dong et al. (2016) on *Panax notoginseng*, which also belongs to *Panax*. The isolated *Fusarium* increased as the American ginseng aged, whereas *Trichoderma* decreased. *Fusarium* is a genus that contains root rot disease causal pathogens (Punja et al., 2005, 2007), and the *Thichoderma* genus contains the most common pathogen antagonists in soil (Vinale et al., 2008; Chen et al., 2011; Gao et al., 2019). The shifts of the two genera in ginseng roots reflect that pathogens become stronger while their antagonism becomes weaker during the growth of American ginseng. In addition, the relative abundance of *Fusarium* spp. in the soil was much higher in the third and fourth years compared with the first 2 years, indicating that the soil environment became less favorable with the increase in the planting years of American ginseng.

Besides, our study found that the occurrences of root rot disease were affected by seasons. As the soil moisture and temperature are quite different among different seasons, the pathogenicity of fungi is different (Wong et al., 1984; Hudec and Muchova, 2010; Guo et al., 2022). In this study, the diversity of bacteria and fungi showed similar seasonal (annual) periodicity during the 4 years of growth of ginseng except for the second year (Figures 2, 4). Similar to our study, Cregger et al. (2012) also found that the variation of microbial communities is highly dependent on seasonal dynamics. Because the microbial community is regulated by climate conditions (e.g., precipitation and temperature), which vary largely between seasons, it is not surprising that the microbial diversity presents a gradient change in different seasons (Guo et al., 2022). Our results revealed that the variation of American ginseng rhizosphere microbial diversity in the second year was not in accordance with the seasonal periodicity. As previously reported, plants can affect the composition of the soil microbial community through plant-soil feedback during growth (Barbara et al., 2015). The soil microbial community should change during rotation. In our study, because the biomass of the maize plant is much larger than that of 1-year-old American ginseng, the effect of maize on the soil microbial community may last until the biomass of American ginseng is large enough to shape the rhizosphere microbial community after 1 year's growth. As a result, the transformation of the maize-shaped microbial community into the American ginseng-shaped microbial community broke the seasonal periodicity in the second year and formed an ecotone of the two communities in the spring of the second year but not the first year, which made the microbial diversity of the 2-year-old ginseng rhizosphere much higher than that in the spring of the other 3 years (Figure 2) because temporal heterogeneity of plant inputs increases soil biodiversity (Eisenhauer, 2016). After the second year, the microbial community finished transforming, thus following the seasonal pattern again in the third and fourth years of American growth. Therefore, the second year is the turning point of new microbial community formation during the 4-year growth of American ginseng. The results of the NMDS analysis also verify the above viewpoint. From the NMDS map, we observed the driving force of the bacterial and fungal community of the American ginseng rhizosphere changing gradually over 1–4 years. The composition of the microbial community in the third and fourth years is close, while that in the second year is between the first year and the last two years (Figure 3). NMDS shows the process of continuous and gradual change in the microbial community under the effect of plants, which is consistent with the previous research on rice (Zhang J. et al., 2018) and cotton (Li D. et al., 2022). Studies have shown that rotation can improve microbial diversity, but the mechanisms are not fully elaborated (Venter et al., 2016; Xie et al., 2022). A potential reason may be that an ecotone of two microbial communities forms when the effects on microbial communities transform from one plant species to another, according to our study.

Proteobacteria, Acidobacteria, and Actinobacteria were the most abundant phyla across all samples, which is consistent with previous studies where results were derived from soils cultivated with maize (Li Y. et al., 2022), American ginseng (Jiang et al., 2019), and even other crops (Shen et al., 2019), indicating that there is no clear distinction among different species of the plant rhizosphere bacteria at the phylum level. At the genus level, *Blastococcus* and *Symbiobacterium* spp. were negatively correlated with DI (Table 2) and decreased over time (Figure 5). *Blastococcus* and *Symbiobacterium* spp. belong to Actinobacteria and Firmicutes, respectively, which were reported to be involved in disease suppression due to the production of biocontrol agents that exhibit antimicrobial effects (Mendes et al., 2011; Cha et al., 2016; Shen et al., 2019). With our findings, we can speculate that Blastococcus and Symbiobacterium spp. may be biocontrol agents of American ginseng, though more research is needed to confirm this. Similarly, *Pandoraea* spp. decreased over time and were negatively correlated with DI in our study, which could suppress pathogens (Jin et al., 2007) and be a potential biocontrol agent (Kotan et al., 2013), as previously reported.

With respect to fungi, our study showed that Ascomycota and Basidiomycota dominated at the phylum level across all samples, which is consistent with a previous study (Shen et al., 2019). Nevertheless, the relative abundance of Ascomycota was positively correlated with the index of disease caused by *Fusarium* in the study of Shen et al. (2019), while some of the fugal genera belonging to Ascomycota were positively correlated with DI, and some were negatively correlated with DI in our study. This discrepancy between the two studies may result from the fact that the taxon is quite different in Ascomycota among different soils, which indicates that microorganisms at the phylum level cannot be used as biomarkers to judge whether their resident soil is conducive to disease or not. Among biomarkers of American ginseng resident time, *Laccaria, Goffeauzyma, Entoloma, Staphylotrichum, Gymnomys, Inocybe, Hirsutella, Penicillium, Tomentella*, and *Suillus* spp. had a significant negative correlation with DI, while *Hebeloma, Elaphomyces, Pseudeurotium, Fusarium, Geomyces, Polyscytalum, Remersonia, Rhizopus, Paraphaeosphaeria, Mortierella*, and *Metarhizium* spp. were significantly positively correlated with the DI of American ginseng. Previous studies about the microbial community and root rot disease of plants of *Panax, Staphylotrichum, Penicillium, Fusarium*, and *Mortierella* spp. were also found to be associated with plant disease (Tan et al., 2017; Jiang et al., 2019; Wei et al., 2020). We also revealed their relationship with resident time to find correlations between DI and fungi. We found that fungi negatively correlated with DI in 1- and 2-year samples, while fungi positively correlated with DI in 3- and 4-year samples. These phenomena can partly explain why the disease in 3- and 4-year-old American ginseng was more severe than that in 1- and 2-year-old American ginseng. Unfortunately, the factors leading to the change in the rhizosphere microbial community are complex and diverse, such as plants (Liu et al., 2022) and nutrients (Rosinger et al., 2022). Thus, to establish a causal relationship between rhizosphere microbial community shift and disease index, more experiments, as described in the study of Zhou et al. (2022), need to be performed in future studies.

When discussing microbial diversity, many researchers believe that a high microbial diversity index signifies healthy soil and can suppress plant disease (Janvier et al., 2007; Larkin, 2015; Vukicevich et al., 2016). In our study, however, the bacterial and fungal Shannon had no significant correlation with DI, while the bacterial and fungal Chao1 were positively correlated with DI. Although it is not common, previous studies also reported a similar phenomenon: the bacterial Chao1 index of disease-suppressive soil was lower than that of disease-conducive soil (Xiong et al., 2017), and the fungal Chao1 showed increasing trends in soils used to cultivate American ginseng compared with those of traditional cropping systems (Dong et al., 2017). Unfortunately, neither of the two studies explained the possible reasons. According to previous studies, when infected by pathogens in soil, plants may recruit many other rhizosphere microorganisms to fight against pathogens (Xiong et al., 2017; Huang et al., 2019), which sometimes contributes to the increase in microbial diversity. To our knowledge, however, the exact mechanism still needs to be elucidated. Microbial diversity may not be a robust index for evaluating whether the soil is disease-suppressive or conducive, especially for agricultural soils, which are disturbed most by human activity.

Moreover, the composition of soil microorganisms is complex, and the diversity index is not competent to reflect the composition of microorganisms. For example, microbial communities with the same diversity may have different richness and evenness (Kennedy, 1999; Hu et al., 2022). In this study, the microbial community structure changed severely during the four years, especially in the second year of American ginseng growth, but the diversity index did not change significantly among the 1-, 3-, and 4-year soils in the same seasons, indicating that a relatively steady microbial structure formed gradually, which can be supported by the study of Jiao et al. (2019) who demonstrated that at least three years' rotation is needed to replant American ginseng.

Soil chemical properties gradually change with plant growth and fertilization in the field (Jiao et al., 2019). In our study, organic matter and pH were higher in 1- and 2-year soil, while AK and AN were higher in 3- and 4-year soil (Table 3), resulting from American ginseng fertilization strategies. In our study and usually in China, farmers apply sufficient organic fertilizer before the sowing of American ginseng, and apply more chemical fertilizer mainly composed of available nutrients in the third and fourth year of growth of American ginseng. Soil chemical properties also affected the soil microbial community (Table 3), which is consistent with previous reports (Bell et al., 2013; Dong et al., 2016). Soil AK and AN were positively correlated with DI, while pH and organic matter were negatively correlated with DI (Figure 6), indicating that the alternation of soil nutrients aggravates the soil microbial community, which makes root rot disease occur more easily. As a result, we recommend using more organic fertilizer and less chemical fertilizer during the third and fourth years of the growth of American ginseng.

## 5. Conclusion

The root rot disease in American ginseng grows more severe with each year that it is cultivated. The second year is the vital period for shifting the American ginseng's rhizosphere microbial community. Disease aggravation after the third year is related to the deterioration of the rhizosphere microecosystem. Increases in soil available nutrients and decreases in organic matter may be related to changes in rhizosphere microbial community composition, diversity, and DI, implying that, in the third and fourth years of American ginseng growth, we should reduce chemical fertilizer input and increase organic fertilizer input.

## Data availability statement

The datasets presented in this study can be found in online repositories. The names of the repository/repositories and accession number(s) can be found in the article/Supplementary material.

## Author contributions

W-WG and Y-MB conceived and designed the research. Y-MB, X-MZ, X-LJ, YW, and J-FL performed the experiments. Y-MB and G-LT analyzed the data. Y-MB, W-WG, and NP wrote the manuscript. All authors read and approved the paper.
