# Supplementary material for: The relationship between shifts in the rhizosphere microbial community and root rot disease in a continuous cropping American ginseng system
Source: Front Microbiol. 2023 Feb 14;14:1097742. doi: 10.3389/fmicb.2023.1097742 (PMC9971623; doi:10.3389/fmicb.2023.1097742)
Supplement: Supplementary file 1 [file Data_Sheet_1.docx]

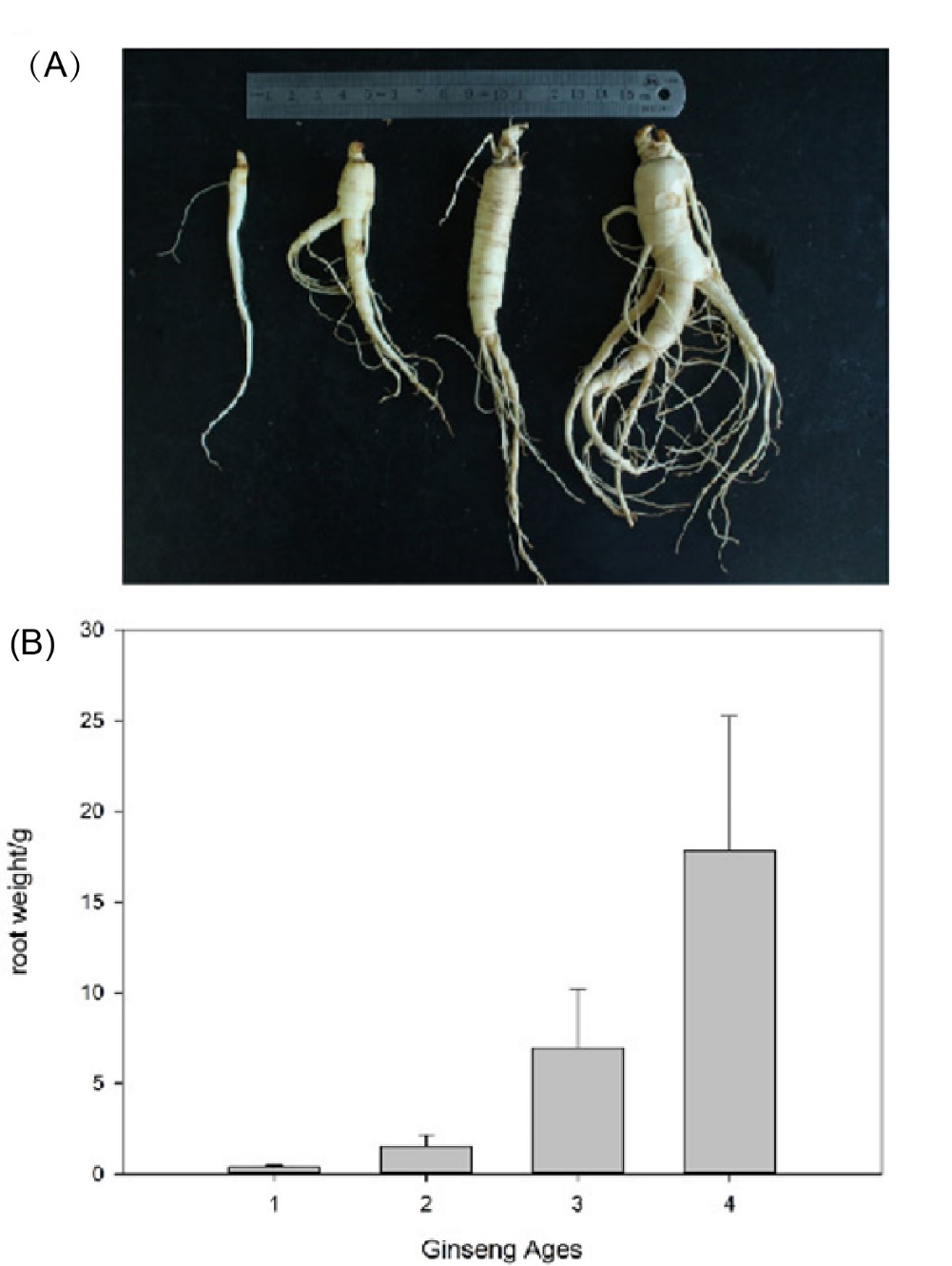


**Supplementary Figure 1.** The root (**A**) and root weight (**B**) of American ginseng of 1- to 4-year.


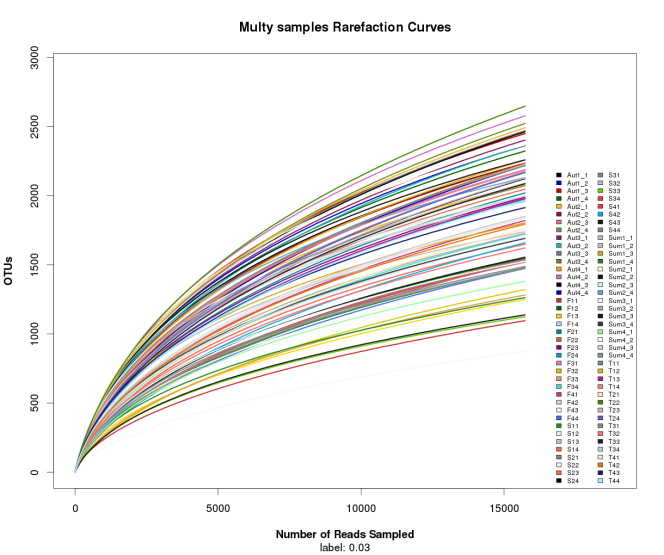

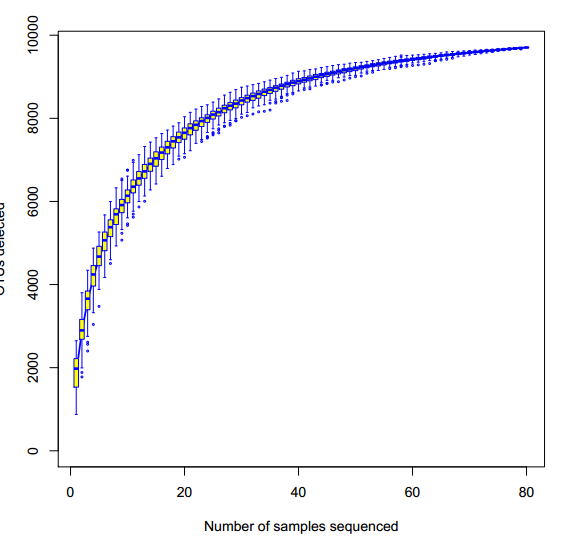


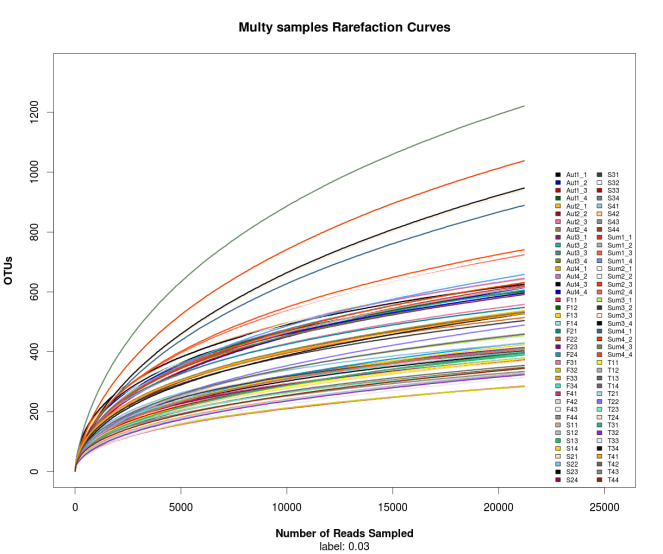

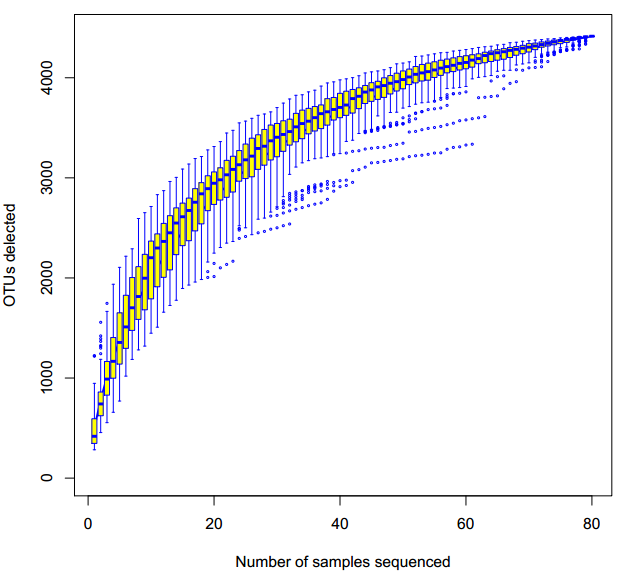


**Supplementary Figure 2.** Rarefaction and species accumulation curves of 16S and ITS.


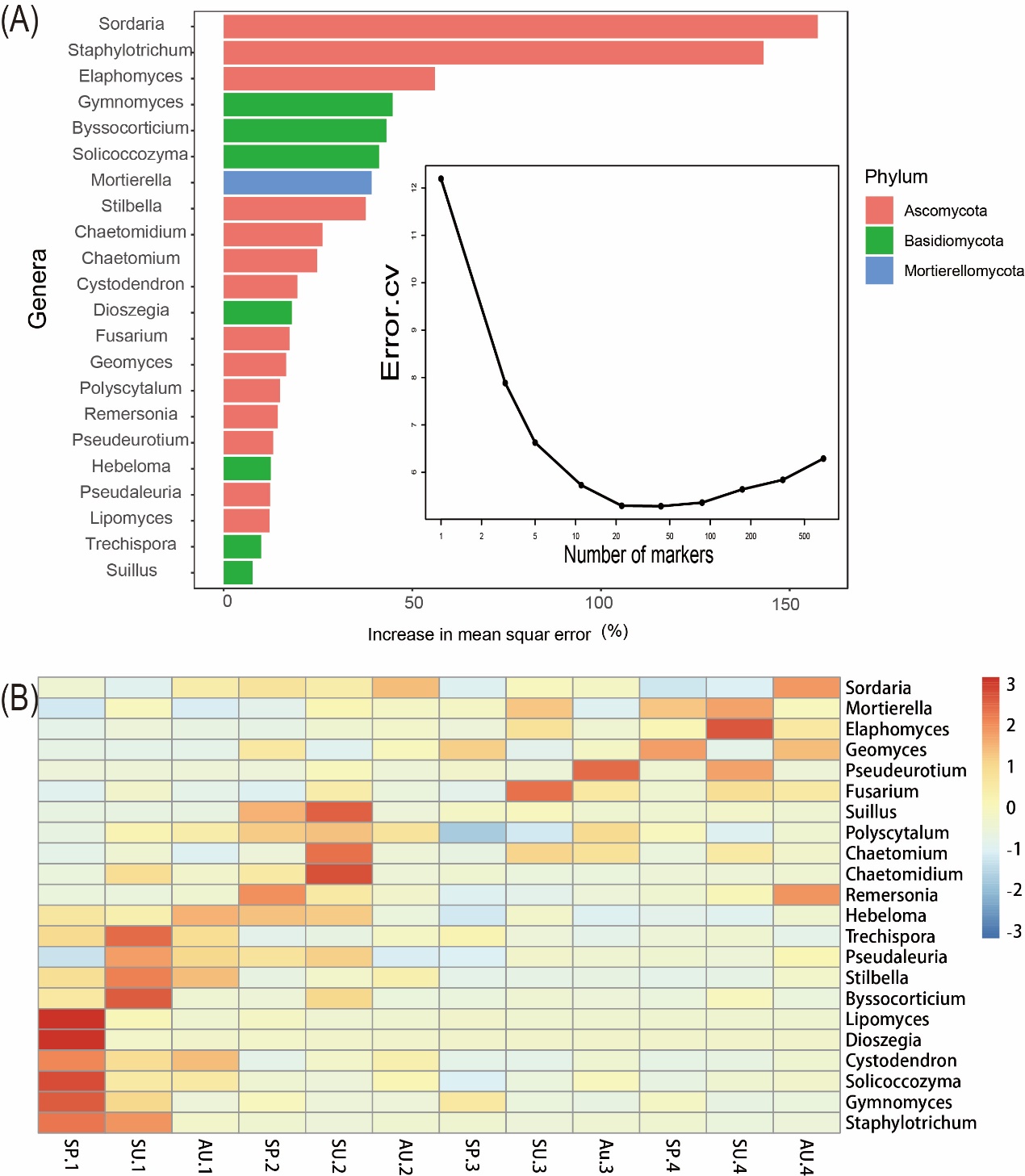


**Supplementary Figure 3.** Fungal taxonomic biomarkers of American ginseng cultivated time in fields (**A**) The top 22 biomarker fungal genera were identified by applying Random Forests regression of their relative abundances in soil against American ginseng cultivated years and seasons in the field. Biomarker taxa are ranked in descending order of importance to the accuracy of the model. (**B**) Heatmap showing the relative abundances of the top 22 predictive biomarker fungal genera.

**Supplementary Figure 4.** Fungi isolated from the diseased root of 1 to 4-year old ginseng.

| Sites | Seasons | Ages | | | |
| --- | --- | --- | --- | --- | --- |
|  |  | 1 | 2 | 3 | 4 |
| Site I | Spring | I_Spr1 | I_Spr2 | I_Spr3 | I_Spr4 |
|  | Summer | I_Sum1 | I_Sum2 | I_Sum3 | I_Sum4 |
|  | Autumn | I_Aut1 | I_Aut2 | I_Aut3 | I_Aut4 |
| Site II | Summer | II_Sum1 | II_Sum2 | II_Sum3 | II_Sum4 |
|  | Autumn | II_Aut1 | II_Aut2 | II_Aut3 | II_Aut4 |

Table S1 Sampling information at two sampling sites of different ginseng ages

Table S2 Mantel test on correlation of soil chemical properties and microbial community

|  | *R* | *P* |
| --- | --- | --- |
| Bacteria | 0.2516 | <0.001 |
| Fungi | 0.3030 | <0.001 |

Table S3 PERMANOVA results for chemical properties and phenolic acids. P-values were calculated based on 999 permutations

|  | Chemical properties | |  | Phenolic acids | |
| --- | --- | --- | --- | --- | --- |
|  | *R^2^* | *P* |  | *R^2^* | *P* |
| Years | 0.1866 | <0.001 |  | 0.4595 | <0.001 |
| Seasons | 0.0761 | 0.004 |  | 0.0681 | 0.003 |
| Years×Seasons | 0.0777 | 0.158 |  | 0.1068 | 0.005 |
